# Datasets from an interaction proteomics screen for substrates of the SCF^βTrCP^ ubiquitin ligase

**DOI:** 10.1016/j.dib.2015.05.003

**Published:** 2015-06-10

**Authors:** Roberto Magliozzi, Mao Peng, Shabaz Mohammed, Daniele Guardavaccaro, Albert J.R. Heck, Teck Yew Low

**Affiliations:** a Hubrecht Institute-KNAW and University Medical Center Utrecht, Uppsalalaan 8, 3584 CH Utrecht, The Netherlands; b Biomolecular Mass Spectrometry and Proteomics, Bijvoet Center for Biomolecular Research and Utrecht Institute for Pharmaceutical Sciences, Utrecht University, Padualaan 8, 3584 H Utrecht, The Netherlands; c Netherlands Proteomics Center, Padualaan 8, 3584 CT Utrecht, The Netherlands

**Keywords:** βTrCP, SCF ubiquitin ligase, F-box protein, Affinity purification-mass spectrometry (AP-MS), Proteomics

## Abstract

An affinity purification-mass spectrometry (AP-MS) method was employed to identify novel substrates of the SCF^βTrCP^ ubiquitin ligase. A FLAG-HA tagged version of the F-box protein βTrCP2, the substrate recognition subunit of SCF^βTrCP^, was used as bait. βTrCP2 wild type and the two mutants βTrCP2-R447A and βTrCP2-ΔF were expressed and purified from HEK293T cells to be able to discriminate between potential substrates of SCF^βTrCP^ and unspecific binders. Affinity-purified samples were analyzed by mass spectrometry-based proteomics, applying ultra-high performance liquid chromatography (UHPLC) coupled to high-resolution tandem mass spectrometry. The raw mass spectrometry data have been deposited to the PRIDE partner repository with the identifiers PXD001088 and PXD001224. The present dataset is associated with a research resource published in T.Y. Low, M. Peng, R. Magliozzi, S. Mohammed, D. Guardavaccaro, A.J.R. Heck, A systems-wide screen identifies substrates of the SCF^βTrCP^ ubiquitin ligase. Sci. Signal. 7 (2014) rs8–rs8, 10.1126/scisignal.2005882.

**Table:** Specifications Table

Subject area	*Biology, Biochemistry*
More specific subject area	*Interaction proteomics studies of an E3 ubiquitin ligase - SCF*^*βTrCP*^
Type of data	(a.) *Raw and processed mass spectrometry data acquired by GeLC–MSMS and LC–MSMS* (b.) *Excel data sheets with identified proteins and peptides*
How data was acquired	(a.) *SDS-PAGE followed by in-gel trypsin digestion and GeLC–MS with Orbitrap Discovery, Orbitrap XL and Orbitrap Velos* (b.) *In-solution trypsin digestion followed by LC–MSMS with Orbitrap Elite*
Data format	*.raw* (*raw mass spectrometry data files*)
	*.xlsx* (*identified peptides and proteins, search parameter with MaxQuant*)
Experimental factors	*Protein samples were reduced with 10* *mM DTT, alkylated with 55* *mM iodoacetamide, digested using sequencing-grade modified trypsin.*
Experimental features	*FLAG-HA tagged βTrCP wild type was expressed in HEK293T cells and used to immunopurify protein interactors via tandem affinity purification* (*TAP*)*. Purified proteins were either analyzed with LC–MSMS directly or after SDS-PAGE separation* (*GeLC–MSMS*)
Data source location	*Biomolecular Mass Spectrometry and Proteomics, Bijvoet Center for Biomolecular Research and Utrecht Institute for Pharmaceutical Sciences, Utrecht University, Padualaan 8, 3584 CH Utrecht, the Netherlands*
Data accessibility	*The mass spectrometry datasets are publicly available from ProteomeXchange Consortium via the PRIDE partner repository*[2] (http://www.proteomexchange.org) *with the dataset identifiers PXD001088* (*LC–MSMS data*) *and PXD001224* (*GeLC–MSMS data*)
	*Results obtained from mass spectrometry post MaxQuant analysis are available as*Supplementary materials*with this paper*

Value of the data

• AP-MS datasets of protein–protein interactions (PPI) for SCF^βTrCP^ ubiquitin ligase.
• These PPIs are validated by two independent statistical platforms.
• A number of proteins bind only to wild type βTrCP and therefore putative substrates.
• Bioinformatics analysis of phosphodegrons supports these substrate candidates.
• Selected candidates are finally confirmed by biochemical experiments.

## 1 Experimental design

**Fig. 1:**
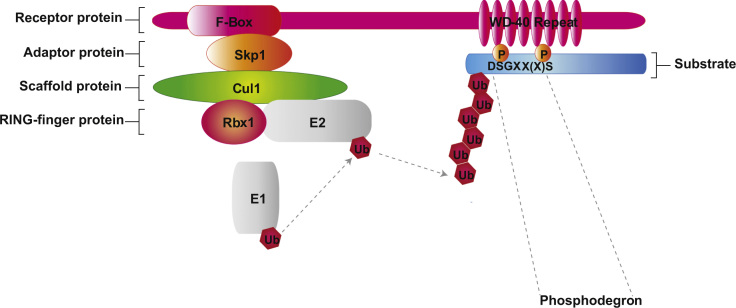
A schematic for SCF^βTrCP^. SCF^βTrCP^ is a multi-subunit E3 ubiquitin ligase consisting of Cul1, Rbx1, Skp1, and the F-box protein βTrCP. Cul1 is a scaffold protein that interacts via its N-terminus with the adapter protein Skp1 and its C-terminus with the RING finger protein Rbx1. Rbx1, in turn binds to a specific ubiquitin conjugating enzyme (E2). Skp1 also interacts with the F-box domain of βTrCP, which recruits substrates through its WD40 repeats. These WD40 repeats form a β-propeller structure, which specifically recognizes a diphosphorylated motif with the consensus motif DpSGXX(X)pS, known as a phosphodegron.

As shown in Fig. 1, SCF^βTrCP^ is a multi-subunit E3 ubiquitin ligase consisting of Cul1, Rbx1, Skp1, and the F-box protein βTrCP. In mammals, two paralogs of βTrCP are present, i.e. βTrCP1 and βTrCP2, however, their biochemical properties appear to be indistinguishable [1]. For our experiments, three different constructs of βTrCP2, each carrying N-terminal 2xFLAG and 2xHA epitope tags were used for independent AP-MS experiments. Epitope tags alone, i.e. without any βTrCP2-based fusion protein (EV), were used as negative control. The three βTrCP2 baits consist of βTrCP2 wild type and two βTrCP2 mutants, i.e. βTrCP2-R447A and βTrCP2-ΔF. The first mutant, βTrCP2-R447A, harbors an Arg to Ala mutation at position 447 of βTrCP2. This substitution within the WD40 β-propeller domain abrogates the interaction with and consequently the ubiquitylation of SCF^βTrCP^ substrates [3,4]. The second mutant, βTrCP2-ΔF, carries a deletion in the F-box motif and acts as a dominant negative mutant [5,6] since it binds substrates but is unable to interact with Skp1, Cul1, Rbx1 and the E2 enzyme.

## 2 Materials and methods

### 2.1 Cell culture, transfection and drug treatment

HEK293T cells were cultured in Dulbecco׳s modified Eagle׳s medium (Invitrogen) with 10% fetal calf serum (FCS) and 100 U ml^−1^ penicillin–streptomycin. HEK293T cells were transfected using the polyethylenimine (PEI) method and, 48 h after transfection, treated with the proteasome inhibitor MG132 (Peptide Institute, 10 μM) for 5 h.

### 2.2 DNA constructs

Full-length, human wild type βTrCP2 (isoform C, NP_036432.2) carrying HA and FLAG tags at the N-terminus was cloned into the EcoRI and NotI sites of pcDNA3 by PCR. The full-length construct was then used as template to generate the βTrCP2-R447A mutant using the QuickChange site-directed mutagenesis kit (Stratagene) according to manufacturer׳s directions.

The following oligonucleotides were used: forward primer:

5′-GGGACATGAAGAATTGGTCGCATGCATCCGGTTTGATAACAAG-3′

and reverse primer:

5′-CTTGTTATCAAACCGGATGCATGCGACCAATTCTTCATGTCCC-3′.

All constructs were verified by sequencing.

### 2.3 βTrCP2 immunopurification

HEK293T cells grown in 15-cm dishes were transfected with pcDNA3-2xFLAG-2xHA-βTrCP2 and treated with 10 μM MG132 for 5 h. Cells were harvested and subsequently lysed in lysis buffer (50 mM Tris–HCl pH 7.5, 150 mM NaCl, 1 mM EDTA and 0.5% NP40). βTrCP2 was immunopurified with mouse anti-FLAG M2 coupled to an agarose resin (Sigma-Aldrich). After washing, proteins were eluted by competition with FLAG peptide (Sigma-Aldrich). The eluate was then subjected to a second immunopurification with anti-HA resin (12CA5 monoclonal antibody cross-linked to protein G Sepharose; Invitrogen).

### 2.4 Recovery of immunoprecipitated proteins for LC–MSMS

For GeLC–MSMS analysis, immunoprecipitated proteins were eluted from the protein G beads with Laemmli sample buffer, separated by SDS-PAGE, and stained with Coomassie colloidal blue. Twelve bands were sliced out from each gel lane which were then reduced, alkylated, and digested according to a published protocol [7]. For direct LC–MSMS analysis, immunoprecipitated proteins on protein G beads were resuspended in a spin filter column (Bio-Rad) and washed three times with 200 μl of phosphate buffer saline to remove residual detergent from the lysis buffer. Proteins bound to the protein G beads were then eluted off with 100 μl of 0.5% RapiGest reagents (Waters) in 50 mM ammonium bicarbonate, followed by 100 μl of 8 M urea in 50 mM ammonium bicarbonate (pH 8.0). Eluted proteins were reduced with 1 mM dithiothreitol (DTT) and alkylated with 5.5 mM iodoacetamide. For tryptic digestion, proteins were first digested with endoproteinase Lys-C (Wako Chemicals) in room temperature for 4 h, followed by sequencing-grade modified trypsin (Promega) overnight, after 4-fold dilution with 50 mM ammonium bicarbonate. Protease digestion was stopped by addition of trifluoroacetic acid (TFA) and precipitates were removed after centrifugation. Peptides were desalted using reversed-phase Sep-Pak C18 cartridges (Waters), then dried and stored at −20 °C.

### 2.5 LC–MSMS

For GeLC–MSMS, we performed nanoflow LC–MSMS with an LTQ-Orbitrap Discovery, Orbitrap XL and Orbitrap Velos mass spectrometers (Thermo Fisher) coupled to an Agilent 1200 HPLC system (Agilent Technologies). Digested peptides were dried, reconstituted in 10% FA and delivered to a trap column (Aqua^™^ C18, 5 µm (Phenomenex); 20 mm×100-µm inner diameter, packed in house) at 5 µl/min in 100% solvent A (0.1 M acetic acid in water). Next, peptides eluted from the trap column onto an analytical column (ReproSil-Pur C18-AQ, 3 µm; Dr. Maisch GmbH; 40 cm×50 µm inner diameter, packed in house) at 100 nl/min in a 1 h gradient from 0 to 40% solvent B (0.1 M acetic acid in 8:2 (v/v) acetonitrile/water). The eluent was sprayed via distal coated emitter tips butt-connected to the analytical column. The mass spectrometer was operated in data-dependent mode, automatically switching between MS and MSMS. Full-scan MS spectra (from *m*/*z* 300 to 1500) were acquired in the Orbitrap with a resolution of 60,000 at *m*/*z* 400 after accumulation to target value of 500,000 in the linear ion trap. The five most intense ions within a survey scan were selected for collision-induced fragmentation (CID) in the linear ion trap at a normalized collision energy of 35% after accumulation to a target value of 10,000.

For direct LC–MSMS, samples were analyzed with Nano-UPLC-MSMS on a Proxeon EASY-nLC 1000 (Thermo Scientific) connected to an LTQ-Orbitrap Elite (Thermo Fisher Scientific). The injected sample was first trapped with a double-fritted trapping column (Reprosil C18, 3 μm, 2 cm×100 μm, Dr. Maisch GmbH) before being separated in an analytical column (Agilent Zorbax SB-C18, 1.8 μm, 35 cm×50 μm). Solvent A consists of 0.1 M acetic acid while solvent B is 0.1 M acetic acid in 80% acetonitrile. Measurement time for each sample took 120 min. Samples are first loaded at a maximum pressure of 980 bar with 100% solvent A. Subsequently, peptides are chromatographically separated by a 91 min gradient consisting of 15–40% solvent B at an un-split flow of 100 nL/min; then ramped to 100% B in 3 min and held in 100% B for another 2 min. This is finally followed by a 13-min equilibration with 100% A. For MS analysis, 1.7 kV was applied to the Nanospray needle. The survey scan was from 350 to 1500 *m*/z at a resolution of 30,000 and for MSMS, the resolution was set to 7500. The 10 most intense precursors were selected for subsequent fragmentation using a direct dependent acquisition. A decision tree method previously described was used [8,9].

### 2.6 LC–MSMS data analysis

MS raw files were analyzed using MaxQuant (version 1.4.0.8, http://141.61.102.17/maxquant_doku/doku.php?id=start) with the match between runs and LFQ options selected. Tandem MS spectra, top 8 selected in 100 Da bin, were searched against the UniProt Human database (Version 2013-07, 20,277 entries). Trypsin/P was chosen as the protease, cysteine carbamidomethylation was set as fixed modification, and oxidation of methionine and acetylation of the N-terminal as variable modifications. Peptide tolerance was initially set to 20 ppm for first search and 4.5 ppm after recalibration, while MSMS tolerance was set to 0.5 Da. All peptide-spectrum matches (PSMs) and proteins were validated with 1% FDR. Only PSMs with a minimum length of 7 amino acids were kept.

### 2.7 Post-acquisition data analysis CRAPome and Perseus analysis

The ‘proteingroups.txt’ table generated by MaxQuant was filtered for contaminants and highly abundant proteins such as keratins, tubulins and ribosomal proteins, reverse hits, number of unique peptides (>0) and number of peptides (>1). Subsequently, analysis and filtering using the CRAPome software suite (www.crapome.org) was performed essentially as described [10]. To discriminate *bona fide* protein interactors of βTrCP2 from the background, we set a SAINT score threshold of 0.9. Subsequently, to identify the putative substrates from this pool of specific interactors, we computed the ratios (FC ratios) for the FC-B scores between the wild type βTrCP2 or βTrCP2-ΔF against the βTrCP2-R447A mutant. For the convenience of FC-ratio estimation, missing values in FC-B scores were imputed with a minimal value of 0.1.

For analysis using the Perseus software suite (http://141.61.102.17/perseus_doku/doku.php?id=start) for identifying specific interactors of wild type βTrCP2 or βTrCP2-ΔF against that of the βTrCP2-R447A mutant, *t*-test-based statistics was applied on LFQ [11]. First, the LFQ values were transformed to logarithm (log 2), and the resulting Gaussian distribution of the data was used for imputation of missing values by normal distribution (width=0.3, shift=2.5). Statistical outliers were then determined using a two-tailed *t*-test followed by multiple testing corrections with a permutation-based false discovery rate (FDR) method. Detailed results for CRAPome and Perseus analyses have been published and are available as Supplementary materials [1] and therefore will not be further described here.

## 3 Data availability

The mass spectrometry datasets are publicly available from ProteomeXchange Consortium via the PRIDE partner repository (http://www.proteomexchange.org) with the dataset identifiers PXD001224 (GeLC–MSMS data) and PXD001088 (LC–MSMS data). Results obtained from mass spectrometry post MaxQuant analysis are available as Supplementary materials with this paper. A list of different types of data made available by this paper is presented as Table 1.

## Funding

This work has been supported by The Netherlands Organization for Scientific Research (NWO) supported large-scale proteomics facility Proteins At Work (project 184.032.201) embedded in the Netherlands Proteomics Centre and by the PRIME-XS project grant agreement number 262067 supported by the European Community׳s Seventh Framework Programme (FP7/2007-2013) to AJRH.

## Figures and Tables

**Table 1:** A list of different types of data made available by this paper.

**Method**	**Sample**	**Separation**	**Replicates**	**No. of RAW files**	**No. of.xlsx files**
GeLC–MSMS (PXD001224)	EV	SDS-PAGE	3	36	4 zipped Excel files generated by MaxQuant:
	βTRCP-WT	SDS-PAGE	3	36	(i.) proteinGroups.xlsx (ii.) peptide.xlsx (iii.) summary.xlsx (iv.) parameters.xlsx
	βTRCP-R447A	SDS-PAGE	3	36	
LC–MSMS (PXD001088)	EV	Direct LC–MSMS	3	3	4 zipped Excel files generated by MaxQuant:
	βTRCP-WT	Direct LC–MSMS	3	3	(i.) proteinGroups.xlsx (ii.) peptide.xlsx (iii.) summary.xlsx (iv.) parameters.xlsx
	βTRCP-R447A	Direct LC–MSMS	3	3	
	βTRCP-ΔF	Direct LC–MSMS	3	3	
